# Supplementary material for: Metal coordinating inhibitors of Rift Valley fever virus replication
Source: PLoS One. 2022 Sep 16;17(9):e0274266. doi: 10.1371/journal.pone.0274266 (PMC9481026; doi:10.1371/journal.pone.0274266)
Supplement: S2 Data — (PDF) [file pone.0274266.s004.pdf]

Supplemental file 2. Structures of compounds in Tables 1 and 2.

α-Hydroxytropolones

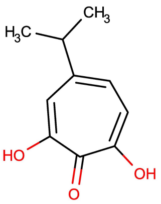

46

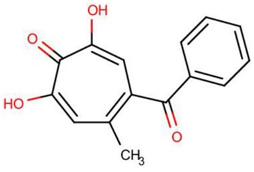

111

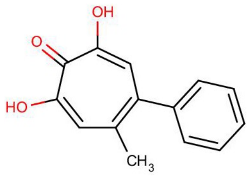

113

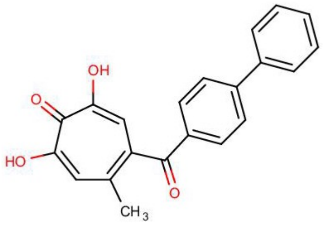

118

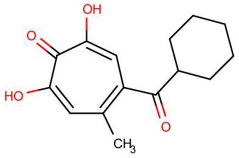

120

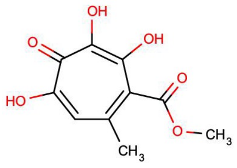

196

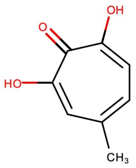

210

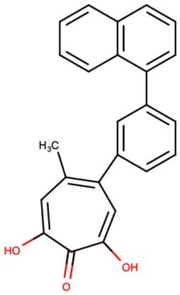

234

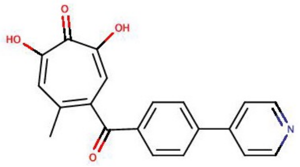

260

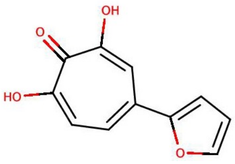

265

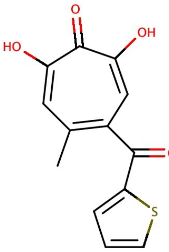

308

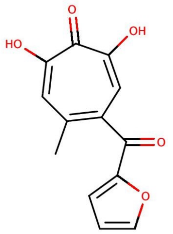

309

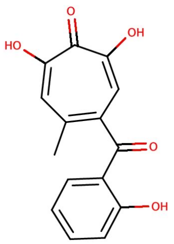

311

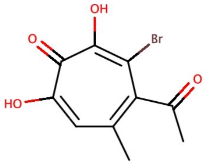

320

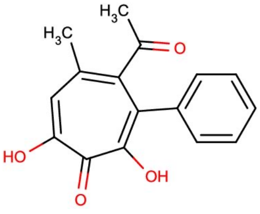

330

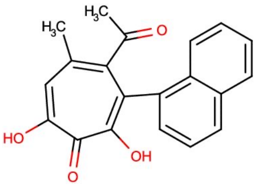

331

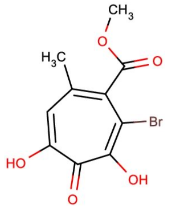

335

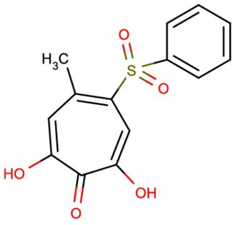

336

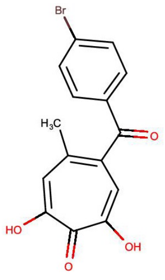

358

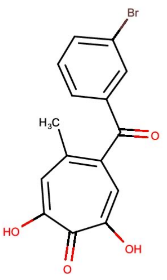

359

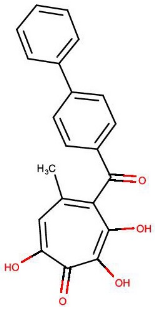

362

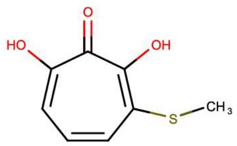

385

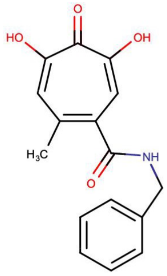

388

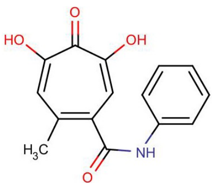

389

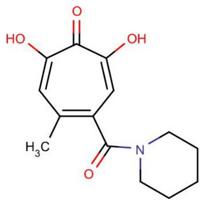

390

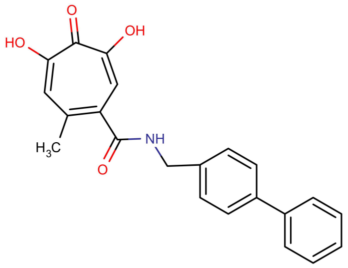

539

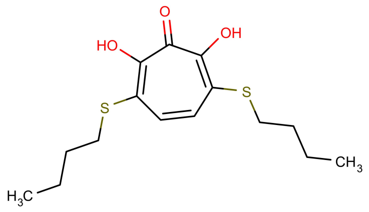

694

<u>α-Hydroxytropolones (Continued)</u>

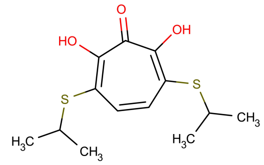

696

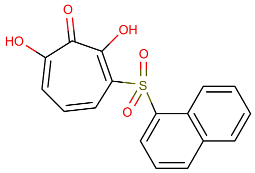

698

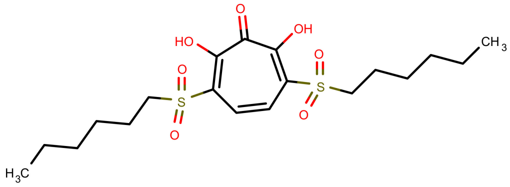

700

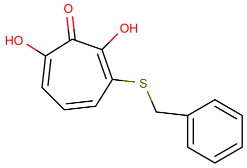

702

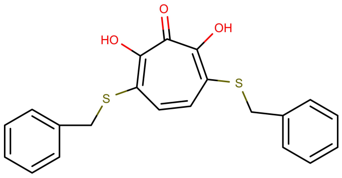

703

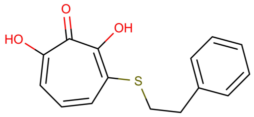

704

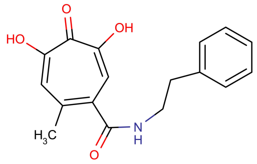

710

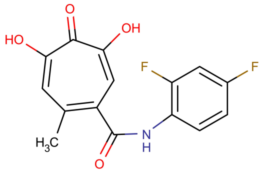

711

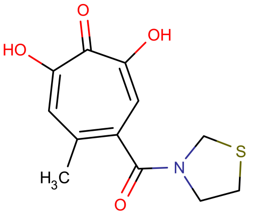

712

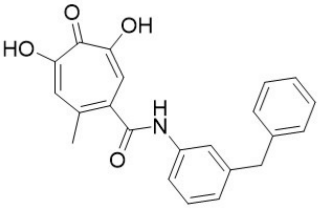

799

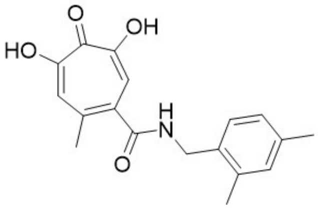

809

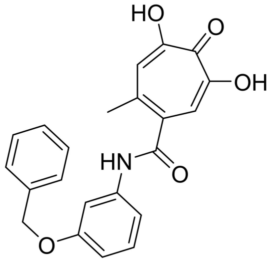

836

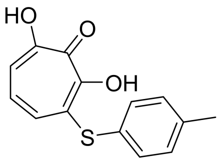

838

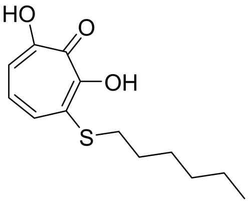

840

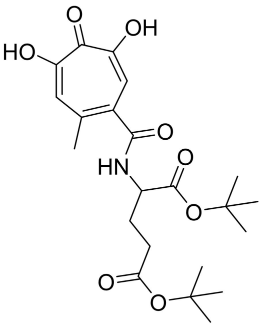

867

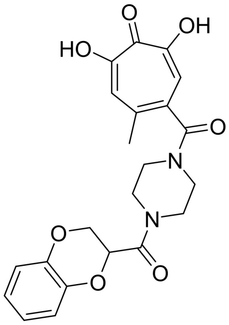

876

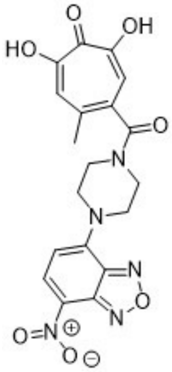

920

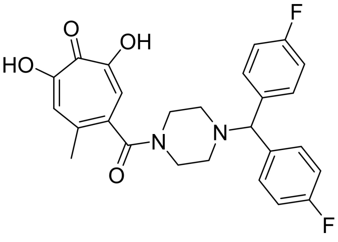

1017

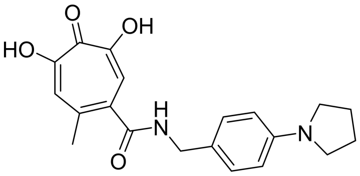

1019

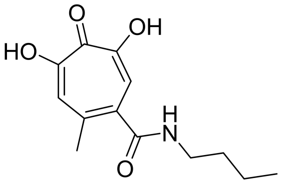

1039

Tropolone and thiotropolones

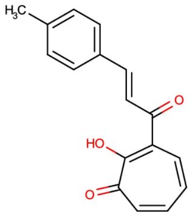

340

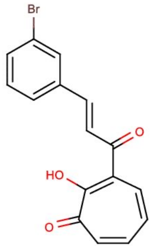

341

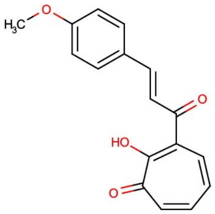

342

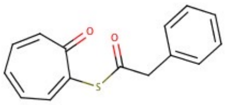

680

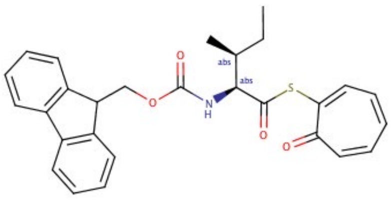

686

N-Hydroxypyridinediones

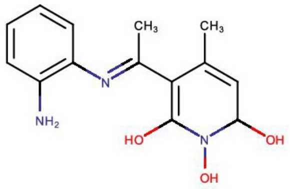

208

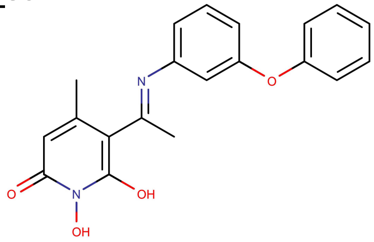

515

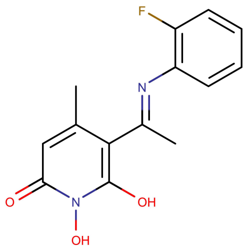

516

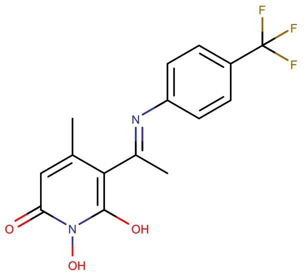

517

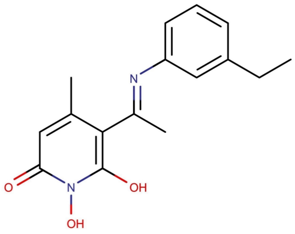

518

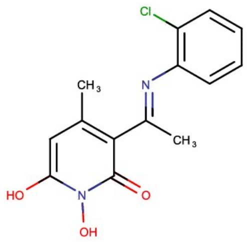

668

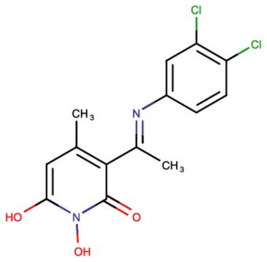

670

Dihydronapthalene

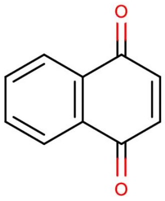

327

Example non-hit compounds

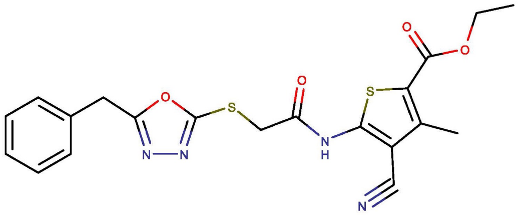

6

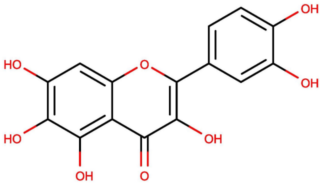

7

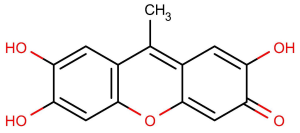

8

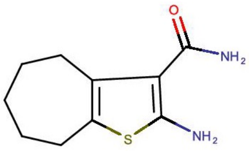

22

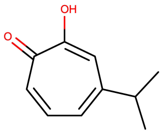

47

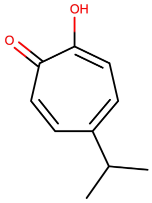

48

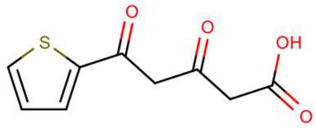

129

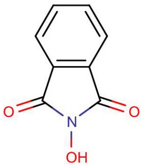

138

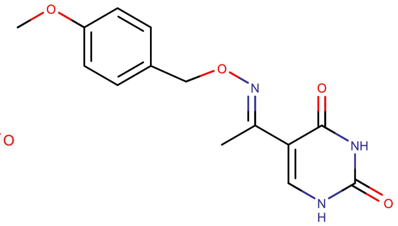

522

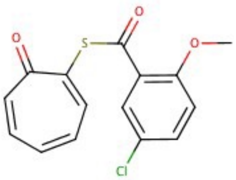

681
