# Supplementary material for: Metal coordinating inhibitors of Rift Valley fever virus replication
Source: PLoS One. 2022 Sep 16;17(9):e0274266. doi: 10.1371/journal.pone.0274266 (PMC9481026; doi:10.1371/journal.pone.0274266)
Supplement: S3 Data — (PDF) [file pone.0274266.s005.pdf]

Supplemental file 3. Confirmed hit compounds.

α-Hydroxytropolones

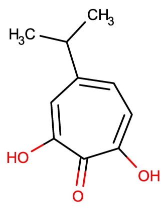

46

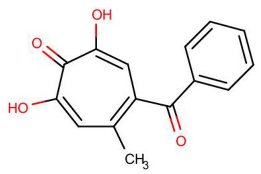

111

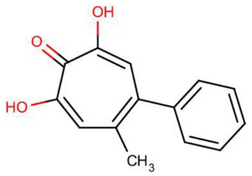

113

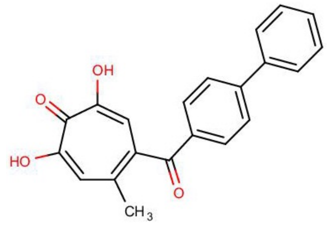

118

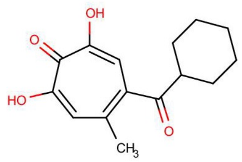

120

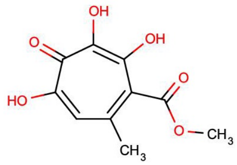

196

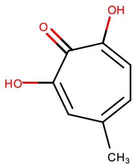

210

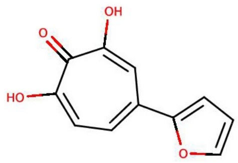

265

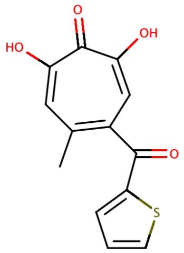

308

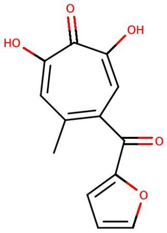

309

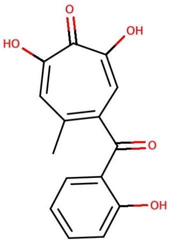

311

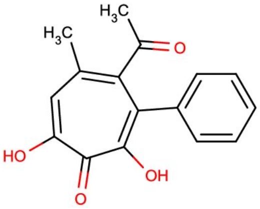

330

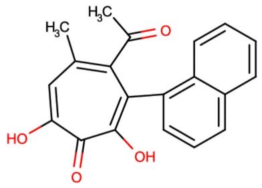

331

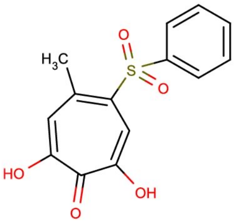

336

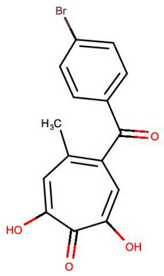

358

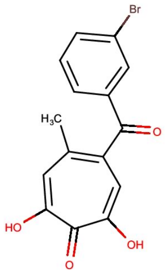

359

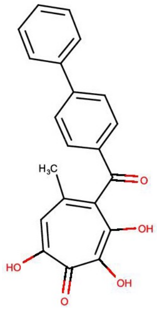

362

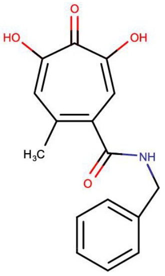

388

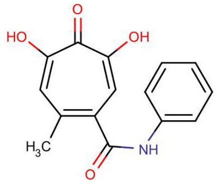

389

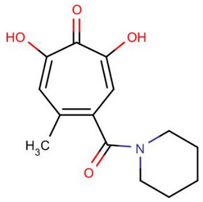

390

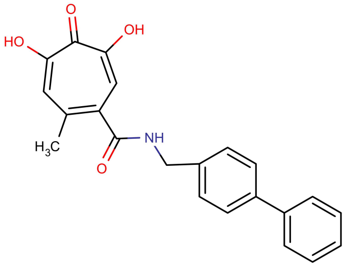

539

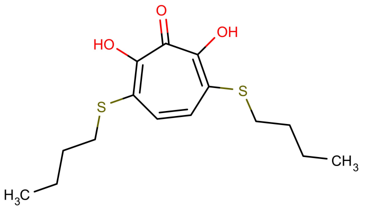

694

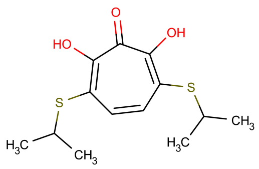

696

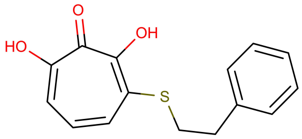

704

α-Hydroxytropolones (continued)

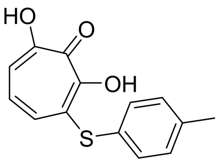

838

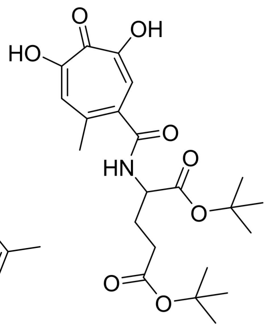

867

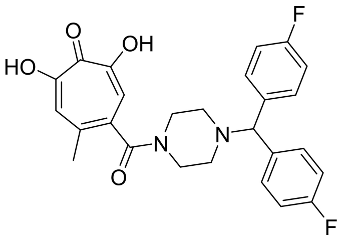

1017

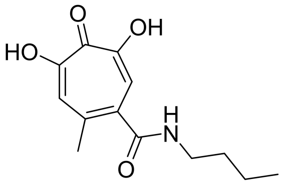

1039

N-Hydroxypyridinediones

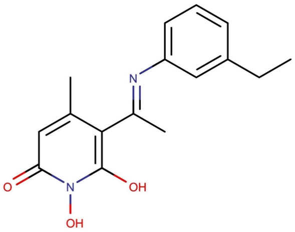

518

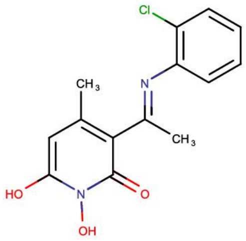

668

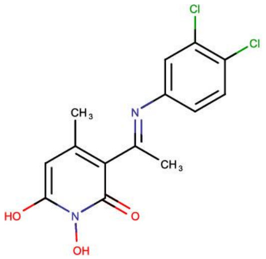

670
